# Extension and Advisory Organizations on the Road to the Digitalization of Animal Farming: An Organizational Learning Perspective

**DOI:** 10.3390/ani10112056

**Published:** 2020-11-06

**Authors:** Chrysanthi Charatsari, Evagelos D. Lioutas, Marcello De Rosa, Afroditi Papadaki-Klavdianou

**Affiliations:** 1 Department of Agricultural Economics, School of Agriculture, Aristotle University of Thessaloniki, 54124 Thessaloniki, Greece; papklavd@agro.auth.gr; 2 School of Humanities, Hellenic Open University, 26335 Patras, Greece; 3 Department of Supply Chain Management, International Hellenic University, 60100 Katerini, Greece; evagelos@agro.auth.gr; 4 School of Social Sciences, Hellenic Open University, 26335 Patras, Greece; 5 Department of Economics and Law, University of Cassino and Southern Lazio, 03043 Cassino, Italy; mderosa@unicas.it

**Keywords:** digital agriculture, animal farming, extension, advisory organizations, digitalization, smart farming, Agriculture 4.0, organizational change, organizational learning, advisers

## Abstract

**Simple Summary:**

While agricultural digitalization is viewed as a revolutionary shift that has the potential to regenerate agriculture, it may have disruptive impacts on agricultural extension and advisory organizations. Adaptive or transitional change (morphostasis) can help these organizations survive and achieve their goals through learning to perform their chosen strategies in a different environment. On the other hand, transformative change (morphogenesis) leads organizations to learn by questioning their purposes, value systems, routines, and operating paradigms, and by moving out of their comfort zone. In this conceptual article, we outline these two change pathways, and we present the learning opportunities that they create for extension and advisory organizations.

**Abstract:**

Agricultural digitalization emerged as a radical innovation, punctuating the gradual evolution of the agrifood sector and having the potential to fundamentally restructure the context within which extension and advisory organizations operate. Digital technologies are expected to alter the practice and culture of animal farming in the future. To suit the changing environmental conditions, organizations can make minor adjustments or can call into question their purposes, belief systems, and operating paradigms. Each pattern of change is associated with different types of organizational learning. In this conceptual article, adopting an organizational learning perspective and building upon organizational change models, we present two potential change and learning pathways that extension and advisory organizations can follow to cope with digitalization: morphostasis and morphogenesis. Morphostatic change has a transitional nature and helps organizations survive by adapting to the new environmental conditions. Organizations that follow this pathway learn by recognizing and correcting errors. This way, they increase their competence in specific services and activities. Morphogenetic change, on the other hand, occurs when organizations acknowledge the need to move beyond existing operating paradigms, redefine their purposes, and explore new possibilities. By transforming themselves, organizations learn new ways to understand and interpret contextual cues. We conclude by presenting some factors that explain extension and advisory organizations’ tendency to morphostasis.

## 1. Introduction

Terms like “digital agriculture” and “smart farming” are today popular in general and scientific vocabularies, whereas the interest in the potential of digital technologies for transforming animal farming is growing apace. In scientific [1,2] and policy documents [3,4], as well as in popular media [5,6], digital farming is viewed as a revolutionary shift, which, by taking advantage of technological sophistication, can increase productivity while reducing the environmental footprint of agriculture. The terms “digital” and “smart” refer to data-rich services and applications, which—supported by advanced hardware—permit farmers and farm managers to smartly analyze, plan, and control farm operations [7]. The process through which these smart innovations penetrate the agrifood sector is termed agricultural digitalization [8].

While, traditionally, farming was based on the process of combining (based on past data, experience, and naked eye observations) natural resources to produce food and fiber, digital farming is driven by real-time (big) data championed by context and situation awareness [9]. Intelligent technologies and big data offer farmers and other actors involved in the agrifood production and supply systems the opportunity to access enormous datasets that collect and store data derived from different sources, thus transforming agriculture from process-driven to data-driven. Such a shift alters the structure of physical-social farming systems, transforming them into cyber-physical-social systems [10].

The cyber dimension of these systems refers to the sum of devices and applications used to collect and analyze data, as well as to the data themselves [9]. The social dimension concerns the web of actors that practice, affect, and are affected by farming (farmers, farm workers, input suppliers, service providers, consumers, etc.). The physical dimension—a term derived from the Greek word φύσις that is used to describe the biotic and abiotic nature and the laws by which it is governed—includes all the tangible elements of a farm (machines, buildings, animal capital, crops) and also their interactions with and within biophysical systems, thus encompassing an ecological sub-dimension (which some scholars refer to as a distinct feature of such systems).

This conversion from a two-dimensional physical-social system to a three-dimensional cyber-physical-social system is a radical leap that can transform farming [11]. Digital technologies present all the main attributes of radical innovation. First, they are based on embryonic but rapidly developing technologies [12]. Undoubtedly, technologies used in digital livestock farming—like big data [13], automatic milking robots [14], virtual fences for controlling livestock [15], convolutional neural network models for identifying animals [16], wearable nano-biosensors [17], microcontroller devices for monitoring the heart rate of animals [18], and digital twins [19]—as well as big data analytics and machine learning algorithms [20,21,22], miniaturized computing devices [23], and genome editing processes [24], are in a continuous and fast evolution process. Second, the adoption of radical innovations is always associated with a high degree of risk [25], which, in the case of digital agriculture, may refer to financial or socio-ethical threats [26,27,28]. Third, when radical innovations arise, new players enter the market to fill critical gaps in its existing structure [29]. Indeed, agricultural digitalization leads to the appearance of many new actors who develop and offer new products or services (such as smart equipment, software and hardware systems, and data analytics) [30,31]. Fourth, the introduction of a radical innovation makes “old knowledge” seem obsolete, thus creating new knowledge needs [32]. Perhaps it goes without saying that digital technologies create the need for new skills associated with both the human–technology interaction [33] and the management of the new “digitalized” organizations [34]. Fifth, radical innovations promote a new modus operandi in the adopting organizations [35] and the actors connected with them through institutional or market linkages [36]. Arguably, digital technologies and big data change the ways farming is practiced, affecting not only farm labor but also the social dimension of agriculture [9,37,38]. In this vein, the digitalization process also affects all the organizations that are variously connected with farming [10].

Looking at extension and advisory organizations, one can see that they have multiple roles to play during agricultural digitalization: they have to act as change agents [39], value extractors [40,41], sense-makers [42], knowledge and information brokers [43]. The new duties that they are expected to perform urge these organizations to alter themselves [44]. New practices, work routines, structures, and organizational arrangements can, and already do, emerge [8,40,45], whereas changes in core organizational attributes (purposes, values) are also possible [46].

From the field of organizational science, we know that, when the external environment changes radically, organizations follow different change pathways, depending on some external—e.g., institutional pressures [47], and positions in networks [48]—and internal—like values, purposes, leadership styles, and organizational biographies [49,50,51,52]—factors. Notably, while changing, organizations assimilate new knowledge through a process known as organizational learning. Simply put, organizational learning is the process through which the organization alters its models of thinking and acting by experiencing internal and/or external changes or discontinuities [53,54,55].

Different patterns of change are associated with different types of learning [56]: organizations can respond to external disturbances either by making minor changes that do not affect their belief and value systems or by questioning their ideologies and their operating paradigms. In the first case, organizations engage in what can be termed single-loop [57], first-order [58], or adaptive learning [54]. This process involves the detection and correction of errors through a conservative, routine procedure that aims at helping the organization to survive by keeping its stability in a changing world and by gaining competence in specific routines or activities [58]. In the second case, organizations do not try to master existing structures and routines or to refine their competencies, but instead explore new paradigms [59]. This type of learning—known as double-loop [57], second-order [58], or generative learning [54]—implies a fundamental transformation since it leads organizations to realize that current paradigms and theories-in-use cannot help them meet their aspirations and that goals, strategies to pursue them, and philosophies should be reconsidered [54,56,58,60].

Shedding light on the different change and learning pathways that extension and advisory organizations follow to cope with the disturbances associated with digitalization can help us estimate their capability in assisting the digital transformation of animal farming. Organizations offering extension and advisory support to livestock farmers have to deal with a variety of challenging issues, including animal welfare, prevention of infectious diseases, complex distribution networks, and changing legislation. In the present article, following an organizational learning perspective, we aim at analyzing the potential change and learning pathways that these organizations can follow to respond to the challenge of digitalization.

## 2. Digitalization and Organizational Change in Extension and Advisory Organizations

### 2.1. Agricultural Digitalization as a Punctuational Change

The introduction of digital technologies in the field of animal farming is a fundamental change, but by no means the first abrupt shift noticed in the field of agriculture. Some scholars [61,62,63] note that digital farming represents the fourth agricultural revolution, which can introduce a new phase in agricultural history, after a relatively long period during which technology continued to develop incrementally. However, despite their potential positive effects, digital technologies have a disruptive and transformational nature, which may shake up the organizations involved in agrifood production and supply systems [61,64,65,66].

In our view, the best way to understand how digital technologies can transform current agrifood architectures is to lean on the knowledge offered by one of the oldest scientific fields: evolutionary biology. Darwin [67], in his famous work, postulated that species evolve through a gradual and cumulative process. However, fossil records seem to reject the gradualism hypothesis, indicating significant gaps in species evolution. To explain such gaps, Eldredge and Gould [68] developed the widely accepted theory of punctuated equilibria. To this theory, long periods of incremental or no change are punctuated by short intervals characterized by radical change. When the environment changes rapidly and radically, species are forced to adjust to the new conditions to survive [69,70].

By adopting such a view—which has a long-term tradition in fields like organizational behavior [58] or management science [71]—one can conceptualize the rise of digital farming as punctuation, leading to the emergence of morphologically diverse organizations [72]. Environmental disturbances urge living organisms to alter themselves in an attempt to survive. This way, species not only avoid extinction but also learn how to react when sudden environmental changes occur. So it is with organizations. While changing to respond to external disturbances, they also learn how to deal with uncertainty and how to cope with external challenges. However, not all organizations follow the same pattern of change. Some initiate minor changes while retaining existing operating processes, whereas some move to new revolutionary models.

### 2.2. How do Extension and Advisory Organizations React to Punctuational Changes?

Extension and advisory organizations are depicted in the literature as socio-economic entities, which combine human resources, financial capital, infrastructures, and equipment to produce services [73] that facilitate agricultural innovation, enhance farmers’ competencies, and promote sustainable rural development [74]. To do so, these organizations create connections with other actors, forming solution networks [75], i.e., constellations of actors who integrate resources to produce solutions to current or future problems [76,77]. This high level of interconnectivity with other actors (farmers, input and technology providers, research institutes, funders, NGOs, etc.) makes such organizations sensitive to external disturbances and punctuations. To understand how extension and advisory organizations might react to the punctuation of agricultural digitalization, we must take a brief look at their organizational anatomy.

From an organizational theory perspective [78], extension and advisory organizations can be viewed as social units composed of people that operate in a relatively continuous way, under the aim of achieving specific sets of goals. These goals—which are not always well-defined—affect organizational missions, which, in turn, are reflected not only in the organizational structure and the services offered (or the provided solutions) but also in the work motifs and the culture that prevail in an organization. Although farmers interact mainly with front-office employees when experiencing the service encounter process [79,80], a set of other attributes, ranging from semi-tangible to purely intangible, defines these organizations (Table 1).

According to Laughlin [81], organizations can be conceived of as amalgams of three different dimensions. The first one refers to the tangible (or visible) elements or organizational sub-systems, which include infrastructures, technologies and artifacts, capitals, employees, and the behaviors of these sub-units. The second refers to organizational structures and management systems, decision-making processes, and communication patterns. These elements constitute what Laughlin and other organizational scholars term “design archetypes.” These archetypes intervene between tangible elements and interpretive schemes (the third—purely intangible—dimension of any organization), i.e., the shared sets of values and beliefs that are based on implicit assumptions about why things are as they are, and what the roles and behaviors of people should be under different situations [82]. Such belief and value systems are used as a compass to develop missions and purposes, which are then expressed in the form of “metarules” or “paradigms” that give direction to the other two dimensions [81]. In other words, interpretive schemes guide the design of organizational archetypes, which set the framework for the operation of organizational sub-systems.

So far, most of the published literature on the characteristics of extension and advisory organizations focuses on the tangible elements of these organizations or their design archetypes [73,74,83], whereas issues associated with interpretive schemes have received only limited attention [84,85]. On the other hand, although scientific work on the ways digitalization impacts these organizations is growing rapidly, there is a lack of studies looking at how the three dimensions of extension and advisory organizations change (if they are doing so) as a response to the digitalization process.

The first systematic attempt to deal with this issue was taken by Rijswijk et al. [46], who, focusing on organizational identity, found that advisory organizations in New Zealand make only some ad hoc changes to adapt to digitalization. This process leads to what organizational change scholars [86,87] term convergent change: the organizations make changes that are minor and consistent with or complementary to operating archetypical templates. Rijswijk and her colleagues [46] operationalized organizational identity as a mixture of tangible and intangible elements, offering some first insights on how sub-systems, design archetypes, and interpretive schemes—albeit not naming them as such—of advisory organizations respond to the effects of the digitalization process.

Nevertheless, research on how these organizations learn when faced with agricultural digitalization is still missing. Although the rise of digital farming spawned a growing interest in the issue of skills that extensionists or advisors should acquire to facilitate the digital transformation of agriculture [11,39,40,42], the focus remains centered on individuals rather than on organizations. However, within organizations, individuals think, act, and interpret different stimuli through the lens of organizational beliefs, frames of reference, and cultures [88,89]. This is a function of organizational learning. As Senge [54] explains, organizational learning depends on but is not the sum or the average of the skills and learning possessed by the individuals within the organization.

Organizational learning is inextricably linked with organizational change. When punctuations occur, organizations engage in adaptive or/and generative learning, which affects the ways employees understand and respond to change. Organizational learning represents a prism through which organizations anticipate their future and the future of their target audience [90], thus deserving special attention.

In the following section, building upon the organizational change models proposed by Laughlin [81] and using an organizational learning lens, we present and discuss two potential types of change and the associated learning pathways that extension and advisory organizations can undergo to adapt to the punctuation caused by the introduction of digital technologies in agriculture.

## 3. Extension and Advisory Organizations in the Time of Digitalization: Change and Learning Pathways

Although the change-promoting character of agricultural digitalization seems indisputable, not all extension and advisory organizations are able to alter themselves or even are expected to endorse the need to change. Change looks like the most logical choice when the external environment is radically transformed since it offers extension and advisory organizations (irrespective of their focus on animal or plant farming) the ability to thrive or at least survive, but inertia remains an option. Inertia practically means doing the same things the same way. In other words, extension and advisory organizations might continue to design and offer the same, well-tested solutions to farmers, rely upon existing structures and management systems, and, most importantly, remain loyal to their belief systems and purposes.

Organizational and management scholars provide a wide array of explanations for inertia: the natural tendency of organizations to keep their status quo [91], the standardization of the procedures used [92], the inability to foresee the need for change [93] and to interpret the environmental cues [94], and the anxiety that the possibility of change generates [95] are just a few of them. Not surprisingly, inertia is also associated with the age and the size of an organization: old and/or large organizations are more likely to remain apathetic during punctuations because of the emphasis that they place on the standardization of processes and control systems, which increases reliability and accountability but, simultaneously, generates resistance to change [96]. Public organizations also have difficulties in overcoming inertia due to the limited levels of pressure they experience from their customers [97].

Irrespective of the reasons behind inertia, organizations that continue to rely on existing ideologies, routines, and practices despite the changes that take place in their environment miss the opportunity to learn, thus losing their ability to cover the changing needs of their customers [98,99] and compromising their own survival [100]. Is this the case for extension and advisory organizations? The lack of studies on the ways these organizations approach digitalization does not permit any conclusion. However, we know that albeit these organizations, especially public ones, may find it hard to follow external changes and to meet new requirements [74], they undertake attempts to follow the external environment [40,101,102]. On the other hand, individual advisors seem to realize that digitalization has the potential to shape a different future for agriculture [103]. However, as we noted earlier, individual perceptions are not enough to explain organizations’ change pathways.

In organizations, structures and management systems are built upon templates of organizing or archetypes [86]. When an organization recognizes that these archetypical templates cannot suit the changing environmental conditions, it may move to new ones. The first critical step in the change process is the realization that the organization cannot sustain its performance (i.e., it is unable to offer efficient solutions to its customers) by reproducing existing structures and activities [104]. This change may have an evolutionary or revolutionary character, depending on the nature of the forces that lead to it [105]. Organizations can initiate incremental changes, thus making minor adjustments that help them keep their position, or they may undertake frame-breaking change.

In the first instance, organizations undergo what Laughlin [81] terms “morphostatic” change: they adapt their design archetypes and some visible elements, without changing their interpretive schemes. Morphostatic change can be temporal (rebuttal change) in the sense that archetypes revert to their previous state when the disruptive force of environmental punctuations decreases, or it can reorient the organization without, however, affecting its core futures. This style of change seems to be what Rijswijk et al. [46] discovered in their study on New Zealand’s advisory organizations (see Section 2.2).

As Table 2 indicates, extension and advisory organizations that choose this pattern of change may alter some of their tangible elements (infrastructures, services) to adapt to the digitalization process. For example, facilitating the adoption of robotic milking technologies can be a new part of the service palette of these organizations. Extensionists or advisors, however, can follow existing routines while designing treatment protocols for cows or when advising farmers on how to reduce the box times. Succinctly put, the organization engages in the practice of doing new things in an old way. Changes in organizational structures are possible depending on the degree to which they are considered as necessary to support the new services, but the central philosophy of the organization remains unchanged.

During morphostatic change, organizations learn by detecting and correcting errors in their practices, within the confines of the operating paradigms in use [106]. This type of learning requires the adoption of existing interpretive schemes and is used to sustain these schemes. The focus is on upskilling, not on the creation of new paradigms. Of course, this does not mean that such type of learning (single-loop, first-order, or adaptive) is of no importance. Through adaptive learning processes, organizations gain mastery over specific activities and services [107] while they increase their efficiency [108] and problem-solving capacity [109], thus adding value to the solutions that they offer to their customers. Hence, extension and advisory organizations that follow a morphostatic change pathway respond to the punctuational change of agricultural digitalization by learning how to better implement their chosen strategies.

A different tactic for these organizations can be to call into question their design archetypes, ideologies, and worldviews, and to initiate and perform alternative operating paradigms. As in the previous case, learning is the response to radical environmental change. However, organizations that follow this pathway learn by moving out of their comfort zone and exploring new possibilities [59]. In this type of learning (double-loop, second-order, or generative), the focus turns from upskilling to relearning. The organization passes through a phase of unlearning—in which the (hard) decision to abandon old design archetypes and interpretive schemes is made and executed—to a new stage, where new knowledge is developed by exploring new paradigms and routines [110]. Generative learning increases organizations’ capacity to change, thus helping them create probable futures [54,111].

Such a change is called morphogenesis because it leads to the transformation of the organization (Table 2). Organizations may initiate morphogenetic change by altering their design archetypes or by first shifting their interpretive schemes. In both cases, the changes penetrate so deeply into the organization that they modify its genetic code [81]. Hence, morphogenesis means much more than just doing new things in a new way. It means understanding and interpreting things in a new way and finding new ways to do new things. It not only offers organizations the opportunity to adapt to current punctuations but also supplies them with the know-how to respond to external disturbances.

Let us use an example to see what morphogenetic change means for extension and advisory organizations. Eastwood et al. [42], based on the findings of their study, suggest that the interpretation of farm data collected and analyzed through digital technologies emerges as a new role for advisory organizations. Farmers need advice on how to translate data analytics into value. An advisory organization that acknowledges the importance of offering such advice might focus on upskilling processes so that advisors can effectively perform the role of “sense-makers.” Alternatively, given that upskilling is not always feasible, it might hire some new employees who already have a level of relevant skills. If the aim continues to be the offering of how-to advice, no changes are expected in organizational belief and value systems. Minor changes to design archetypes will follow the necessary changes in organizations’ tangible elements. That is a morphostatic change.

If, on the other hand, the organization decides to offer integrated solutions to farmers through, for instance, the development of tailor-made digital applications or the offering of algorithmic services—either by establishing a new department or by building alliances with Ag-Tech companies—then the change is morphogenetic. Such a transformation requires major shifts in purposes and rationales, and urges the organization to change its work routines. Both design archetypes (the ways business is done) and interpretive schemes (the ways the organization conceives of digitalization and anticipates its role in it) are called into question, and the organization moves to a new culture.

So, the question emerges: what change pathways should extension and advisory organizations follow to respond to the challenge of digitalization? Morphogenetic change offers organizations the opportunity not to follow but to shape agricultural digitalization, while in parallel transforming them into learning organizations. It increases their ability to cope with the present needs of farmers and enhances their future adaptability to the external environment. Nevertheless, morphogenetic change is not a panacea.

Although, as researchers, we may find the idea of morphogenetic change more intriguing, organizations have a variety of reasons to prefer changes that are consistent with existing interpretive schemes and operating paradigms. Morphogenetic change is always accompanied by a degree of risk and potential costs that sometimes exceed the expected benefits [112]. When uncertainty or resource constraints make the initiation of frame-breaking changes difficult, morphostasis is a wiser strategy. On the other hand, agricultural digitalization is expected to be a lengthy and non-uniform process because of the high heterogeneity of both digital technologies [113] and farming systems [114]. Hence, morphostatic change, albeit more trivial and perhaps mundane, is considered a safe choice because organizations believe that minor adaptations are enough to keep them effective at least in the short term. Besides, during morphogenetic change, organizations encounter practical difficulties. Often, while changing, the organization passes through a “schizoid” phase: an unwanted situation in which different interpretive schemes co-exist, leading to tension and causing operational problems [115]. Finally, organizations that successfully serve their customer base may face considerable difficulties in altering themselves. For them, changing purposes, value systems, and ideologies means calling into question their own success story [116].

## 4. Conclusions

This work, building upon theories of organizational change and organizational learning, is one of the first attempts to paint a picture of the change and learning pathways that extension and advisory organizations can undergo to deal with agricultural digitalization. Digitalization punctuates the gradual evolution of farming and radically alters the agrifood sector, urging the involved actors to change. Under such conditions, inertia is not a prudent choice. Evolutionary biology offers crystal-clear lessons on the future of organisms that resist or are unable to change. Inert organizations might have the same fate.

Hence, transition and transformation are the two options available to extension and advisory organizations. However, the answer to the dilemma “morphogenesis or morphostasis?” cannot be given clearly. Empirical research is needed to illuminate how extension and advisory organizations experience transition (morphostatic) or transformation (morphogenetic) pathways, what internal and external factors facilitate or inhibit organizational change, and the ways these pathways are reflected in farmers’ ability to cope with digitalization. We hope that this work provides some theoretical foundation for future empirical investigations.

## Figures and Tables

**Table 1:** Dimensions of organizations (based on Laughlin’s [81] conceptualization).

Dimension	Elements
Tangible elements (sub-systems)	Infrastructures, technologies, artifacts, people, finance, services, behaviors
Design archetypes	Structure of the organization, decision processes, management systems, communication processes
Interpretive schemes	Norms, belief and value systems, shared assumptions, organizational missions, purposes, paradigms

**Table 2:** Effects of morphostatic and morphogenetic changes on the dimensions of organizations.

Dimension	Morphostasis	Morphogenesis
Tangible elements (sub-systems)	Change in infrastructures, new technologies, new services, and/or new employees	New services, changes in infrastructures and technologies used, formation of inter-organizational collaborations
Design archetypes	Minor changes in organizational structures and communication processes	Major changes in organizational structures, management systems, and decision and communication processes
Interpretive schemes	No change	Deep change in belief and value systems, reconsideration of assumptions, major changes in operating paradigms and missions
